# Engineering single-atom dynamics with electron irradiation

**DOI:** 10.1126/sciadv.aav2252

**Published:** 2019-05-17

**Authors:** Cong Su, Mukesh Tripathi, Qing-Bo Yan, Zegao Wang, Zihan Zhang, Christoph Hofer, Haozhe Wang, Leonardo Basile, Gang Su, Mingdong Dong, Jannik C. Meyer, Jani Kotakoski, Jing Kong, Juan-Carlos Idrobo, Toma Susi, Ju Li

**Affiliations:** 1 Department of Nuclear Science and Engineering and Department of Materials Science and Engineering, Massachusetts Institute of Technology, Cambridge, MA 02139, USA.; 2 Research Lab of Electronics (RLE), Massachusetts Institutes of Technology, Cambridge, MA 02139, USA.; 3 University of Vienna, Faculty of Physics, Vienna 1090, Austria.; 4 College of Materials Science and Opto-Electronic Technology, University of Chinese Academy of Sciences, Beijing 100049, China.; 5 Interdisciplinary Nanoscience Center (iNano), Aarhus University, Aarhus 8000, Denmark.; 6 College of Materials Science and Engineering, Sichuan University, Chengdu 610065, China.; 7 School of Physical Sciences, University of Chinese Academy of Sciences, Beijing 100049, China.; 8 Department of Physics, Escuela Politécnica Nacional, Quito 170517, Ecuador.; 9 Kavli Institute for Theoretical Sciences, and CAS Center of Excellence in Topological Quantum Computation, University of Chinese Academy of Sciences, Beijing 100049, China.; 10 Department of Electrical Engineering and Computer Science, Massachusetts Institute of Technology, Cambridge, MA 02139, USA.; 11 Center for Nanophase Materials Sciences, Oak Ridge National Laboratory, Oak Ridge, TN 37831, USA.

## Abstract

Atomic engineering is envisioned to involve selectively inducing the desired dynamics of single atoms and combining these steps for larger-scale assemblies. Here, we focus on the first part by surveying the single-step dynamics of graphene dopants, primarily phosphorus, caused by electron irradiation both in experiment and simulation, and develop a theory for describing the probabilities of competing configurational outcomes depending on the postcollision momentum vector of the primary knock-on atom. The predicted branching ratio of configurational transformations agrees well with our atomically resolved experiments. This suggests a way for biasing the dynamics toward desired outcomes, paving the road for designing and further upscaling atomic engineering using electron irradiation.

## Teaser

A vector-space formalism is developed for optimizing single-atom manipulation outcomes under focused electron irradiation.

## INTRODUCTION

Controlling the exact atomic structure of materials is an ultimate form of engineering (*1*, *2*). Atomic manipulation and atom-by-atom assembly can create functional structures that are hard to synthesize chemically (*3*–*6*), e.g., exactly positioning atomic dopants to modify the properties of carbon nanotubes and graphene (*7*). Nitrogen (N) or phosphorus (P) dopants might be useful in quantum informatics due to nonzero nuclear spin (*8*).

Successful atomic engineering requires understanding of two parts: (i) how the desirable local configurational changes can be induced to increase the speed and success rate of control and (ii) how to scale up basic unit processes into feasible structural assemblies of 1 to 1000 atoms to produce the desired functionality. Historically, scanning tunneling microscopies (*9*) have demonstrated good stepwise control of single atoms, leading to physicochemical insights and technological advances (*10*). However, their scalability and throughput are severely limited by the mechanical probe movements. Recently, aberration-corrected scanning transmission electron microscopy (STEM) has emerged as a versatile tool for characterizing the precise atomic structure of materials (*11*–*18*). Despite its very early stage of development, STEM also shows great promise as a tool for atomic manipulation: In two-dimensional (2D) graphene, Si dopants are found to be stepwise controllable (*19*–*21*), and iterating these basic steps enables long-range movement with a high throughput (*22*), whereas in a 3D silicon crystal, the projected location of Bi dopants was also manipulated (*23*). However, imprecise understanding of the dynamics of the basic steps, which involves relativistic electron-nucleus collisions, electronic excitation and relaxation, dynamic ion trajectories, momenta dephasing, and heat conduction, add uncertainties to this technique. While the traditional theory of radiation damage provides a basis for understanding, instead of trying to minimize beam effects, atomic engineering seeks to utilize them to achieve desired configurational changes. Concepts like the displacement threshold energy *E*_d_, which is, in most cases, approximated as scalar, turn out to be too crude to guide the design of the precise cross-sections of different configurational outcomes (*24*, *25*).

Here, we use STEM to both drive and identify the atomic motion of individual phosphorus (P) dopants in graphene and construct a theoretical scheme for evaluating their relative probabilities with respect to electron energy and momentum direction. We have categorized the dynamics into four types: (A) direct exchange and (B) Stone-Wales (SW) transition, which conserve the atoms, and (C) knockout of a carbon neighbor and (D) replacement of the dopant atom by C, which do not conserve the local composition. We choose to use ${\tilde{E}}_{e}$ = 60 keV electron energy (velocity ${\tilde{v}}_{e}=$ 0.4462*c* = 1.3377 × 10^8^ m/s) to minimize (C) and (D) while maximizing the rates of direct exchange and SW transition. Hereafter, the variables with tilde (~) on top represent quantities before atom-electron collision, and variables without tilde represent those after collision. Furthermore, instead of aiming the electron beam directly at the dopant itself, it has been established that dynamics can be more effectively induced when the electron beam is aimed at a carbon neighbor of the dopant (*3*, *21*).

To achieve atomic configurational change, the postelectron collisional energy of the primary knock-on atom (PKA; here, it is carbon), *E*, needs to be on the order of 10 eV. This requires the penetrating electron to pass very close to the PKA nucleus (impact parameter *b < b*_c_ ~ 10^−14^ m), with corresponding collisional cross section on the order of barns (σ ~ 10^−28^ m^2^). Such elastic collision and large energy transfer occur mainly within 0.1 zeptosecond time scale (τ_c_ ~ 10^−22^ s), inducing a postcollisional PKA momentum labeled by a vector (θ, φ, E). With a total beam current of *I* ~ 50 pA, this amounts to about one relativistic electron penetrating the graphene every 3 ns (τ_p_ ≡ *e*/*I* ~ 3 ns), and one can focus the e-beam to a spot with a full width at half maximum (FWHM) of ~1 Å (which provides a sufficient description of the scanning beam). The collisional probability (defined as imparting the PKA with *E* ~ 10 eV energy, which may cause “immediate” configurational change within picoseconds) is thus only ~σ/FWHM^2^ ~ 10^−8^ per penetration event or σ/FWHM^2^/τ_p_ ~ 10 per s (0.1 per s for events like direct exchange with cross section of 0.01 barn); the rest of the penetration events cause electronic excitation and small ionic rattling but not immediate local configurational change.

Regardless of whether a penetrating electron gets within *b*_c_ or not, a penetration event will cause electronic excitation, occurring with an attosecond time scale τ_e_ ~ 3.4 Å/${\tilde{v}}_{e}$ ~ 10^−18^ s (3.4 Å being the graphene thickness), which, however, in the case of graphene, will relax collectively on the femtosecond time scale (τ_E_ ~ 10^−15^ s) to the electronic ground state (*26*). Thus, after τ_e_ + τ_E_, the electronic subsystem falls back to electronic equilibrium, one may use the Born-Oppenheimer (BO) approximation to describe the ion dynamics, which can achieve either one of the (A) to (D) configurational changes (labeled by *i* = 1 to 4) or remain unchanged (*i* = 0) on the BO surface, within a few picoseconds (τ_I_ ~ 10^−12^ s). Since τ_I_ > > τ_e_ + τ_E_, it is justified here to apply ground-state density functional theory (DFT) to track the main portion of the ion dynamics and to obtain the probability of success, *P_i_*, of atomic dynamics that lead to configurational outcome *i*.

Throughout *t* = τ_c_, τ_E_, τ_I_, the PKA momentum history needs to be tracked; thus, we build a theoretical scheme called primary knock-on space (PKS) for estimating the relative scattering cross sections of different electron-induced dynamics due to either sample or electron beam tilt and for selectively activating the desired outcomes. We further provide experimental verification of our calculations, thus opening new avenues for atomic engineering using focused electron irradiation.

## RESULTS

We find that the P dopant in graphene can serve as a good example for covering many categories of electron-induced dynamics. With highly collimated and focused (e-beam FWHM ~1 Å) electron irradiation on a carbon atom neighboring the phosphorus dopant, we occasionally create a single energetic PKA, with rate ~*d*σ/FWHM^2^/τ_p_, where *d*σ is the differential cross section corresponding to a particular postcollisional PKA differential momentum volume. To clarify, the term PKA here exclusively refers to the energetic carbon neighbor of the phosphorus dopant, so “PKA” and “C neighbor hit by an electron of the beam” are equivalent throughout this paper. This energetic carbon atom then drives a short burst of atomic motions nearby within τ_I_ ~ picoseconds.

**Fig. 1:**
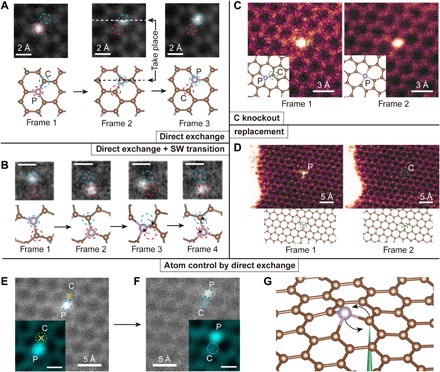
Illustration of competing experimental P dopant dynamics in graphene and its control. The frames are medium-angle annular dark-field images, and the chemical identity of each dopant was confirmed by electron energy-loss spectroscopy (EELS). (**A**) Three frames showing a direct exchange between the brighter (due to its greater scattering contrast) P atom and a C neighbor, with the initial (frame 1), transition (frame 2), and final configurations (frame 3). White and black dashed lines indicate the row of the scanning beam when the exchange happens. Scan speed, 8.4 s per frame. No postprocessing was done. (**B**) Four frames showing both direct exchange (frames 1 and 2) and SW transition (frames 2 to 4). Scale bars, 2 Å. Scan speed, 0.07 s per frame. A median filter with a 2 pixel × 2 pixel kernel was applied for clarity. The SW transition was captured during EELS acquisition in small subscan windows to enhance the signal-to-noise ratio of the spectra used to identify the dopants and to achieve faster scanning rate frames that can better capture atomic dynamics. (**C**) Neighboring C atom knocked out by the electron beam, turning a threefold-coordinated P into fourfold-coordinated P. Scan speed, 8 s per frame. No postprocessing was done. (**D**) P dopant being replaced by a C atom. Scan speed, 4 s per frame. The different image color codings represent different categories: gray represents atom-conserving process, and magenta represents atom-nonconserving process. Blue and red dashed circles in (A) and (B) represent the inequivalent lattice sites of graphene, and the green dashed circles in (C) and (D) indicate the location of the atom that has not been conserved. (**E** and **F**) Intentional control over the P direct exchange. The yellow crosses indicate the location where the electron beam was parked for 10 s to purposefully move the P atom by one lattice site. Green and blue dashed circles indicate the two nonequivalent lattices sites of graphene. Insets: The region of interest after applying a Gaussian filter. (**G**) A schematic plot of the control process, where the electron beam is represented by a green cone focused on the neighbor C atom.

In Fig. 1, four types of dynamics are shown and categorized into two groups: atom-conserving dynamics (which is desirable) and atom-nonconserving dynamics (which is often not desirable). Atom-conserving dynamics include (A) direct exchange between P and C [(Fig. 1A), earlier dubbed “bond inversion” in the context of Si (*21*)] and (B) SW transition (Fig. 1B), i.e., 90° rotation of a P─C bond (*25*). Atom-nonconserving dynamics include (C) knockout (Fig. 1C), where the PKA is knocked out by the electron beam (P turns from three fold-coordinated to four fold-coordinated, after which we found that it is no longer possible to further manipulate the configuration with 60-keV e-beam) and (D) replacement (Fig. 1D), where a diffusing carbon adatom that happens to be nearby receives energy from a penetrating electron and replaces the dopant atom, which then diffuses away quickly. These wandering C adatoms are always present on graphene surfaces (*21*, *27*), but they diffuse too quickly to be imaged. In the above experiments, we scanned the beam over a square area covering the dopant atom so that the configurational changes could also be captured in frames (often as a broken “transit” frame, where part of the scanned image is discontinuous with the rest of the image that is scanned later).

In Fig. 1A, three consecutive frames of direct exchange including a transition frame are presented. As a result, the P dopant atom exchanges site with the PKA, while the e-beam is scanning from left to right across the PKA (white dashed line; note that, at each pixel, most of the electron dose is distributed within an ~Å-sized area surrounding it according to the beam intensity profile). In Fig. 1B, a SW transition is preceded by a direct exchange. After the direct exchange (frames 1 to 2), the P─C bond is rotated by 90° (frames 2 to 3), and the hexagonal lattice is locally transformed into two pairs of five- and seven-membered rings (55-77 structure hereafter). The 55-77 structure is only stable for less than 0.2 s before reverting back to hexagons (frames 3 to 4) due to the subsequent electron irradiation. In Fig. 1C, a threefold-coordinated P (frame 1) turns into fourfold-coordinated P (frame 2) when the PKA is knocked out by the electron beam. Once this happens, we find that the P can no longer be manipulated. In Fig. 1D, P is replaced by C, and then diffuses away, which is the most commonly observed outcome of P impurities—in stark contrast to Si, which are almost never removed or replaced. It should be noted that we never observed a phosphorus being simply knocked out leaving a vacancy behind, consistent with the prediction that its knockout cross section is several orders of magnitude smaller than that of the lighter C atoms.

As a basic test of controlling the P dopant for atomic engineering, a direct exchange is intentionally initialized by targeting the highly focused e-beam at a neighboring C atom. Since the out-of-plane dynamics of the energetic C neighbor are responsible for the change in the structure (*21*), the outcome of the exchange can be controlled by selecting the PKA among the three possible carbon neighbors. The initial position of the P dopant is shown in Fig. 1E. The yellow crosses indicate where the electron beam is parked for 10 s, and afterward, a second frame is immediately captured, as shown in Fig. 1F. As a result, the P atom hops to the site as directed, but this occurred only after 68 ineffective 10-s spot irradiations (another P jumped after 12 10-s spot irradiations). Compared to Si impurities, P is much harder to induce direct exchange with (Fig. 1A): Irradiating the neighbor C site typically triggers the replacement process (Fig. 1D) instead. We tried to manipulate 10 P impurities, 2 of which exchanged (outcome A or B), 1 lost a C neighbor (outcome C), and 7 were replaced by freely diffusing carbon adatom (outcome D) after, on average, 22 ± 5 (mean ± SE) 10-s spot irradiations. Note that because the electron beam is not scanning continuously, we cannot resolve SW transition, which was actually found to have the largest cross-section (Table 1) when we later scanned the beam, since the 55-77 metastable configuration is quite beam-sensitive.

To reduce the replacement of the dopant by freely diffusing C adatoms, we also used double-layer graphene (fig. S1), where atom diffusion on one side is suppressed. It is interesting to observe that the phosphorus dopant in a double-layer graphene is much less likely to be replaced than in single-layer graphene. With a similar dose rate, the P atom was not replaced during our observation (~12 min), which is more than four times longer than in single-layer graphene (~3 min). It should be noted that the difficulty of manipulating P atoms represents a generic challenge in atomic engineering, where a desired configurational outcome is disrupted by other unwanted ones. Our work is specifically focusing on dealing with this issue.

To explain these processes, we have performed extensive ab initio molecular dynamics (abMD) and climbing-image nudged elastic band (cNEB) calculations. With a clear separation of time scales, in particular, τ_E_ < <τ_I_, it is a reasonable approximation to simulate the configurational change processes on the BO surface, assuming each dynamic step evolves according to the Hellman-Feynman forces calculated on the basis of the electronic ground state.

**Fig. 2:**
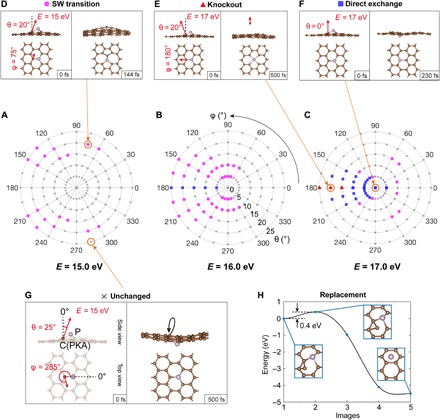
Mechanisms of P dopant dynamics in graphene calculated with abMD. (**A** to **C**) Angular distribution maps of different possible lattice transformations obtained when a C neighbor of the P impurity is given an initial out-of-plane momentum. The corresponding initial kinetic energies on the carbon, *E*, are (A) 15.0, (B) 16.0, and (C) 17.0 eV. The marks in these polar plots indicate the dynamical outcome: C knockout as red triangles, direct exchange as blue squares, SW transitions as magenta circles, and unchanged lattice as black crosses. As examples, snapshots of (**D**) SW transition (θ = 20°, φ = 75°, *E* = 15.0 eV), (**E**) C knockout (θ = 20°, φ = 180°, *E* = 17.0 eV), (**F**) direct exchange (θ = 0°, *E* = 17.0 eV), and (**G**) unchanged structure (θ = 25°, φ = 285°, *E* = 15.0 eV) are shown. The red arrows indicate the direction of the C momentum along the in-plane and normal-to-plane directions (lengths not to scale), with the definition of the spherical coordinate angles θ and φ shown in (G). (**H**) cNEB barrier for a proposed mechanism of P dopant replacement by C. Insets: The initial, saddle-point, and final configurations.

The distribution of various types of dynamics is shown in Fig. 2 (A to C), which corresponds to initial postcollision kinetic energies of the PKA at *E* = 15, 16, and 17 eV, with the angular space sampled with an interval of 15° for the azimuthal angle φ and 5° for the polar angle θ (up to 25°). Figure 2 (D to G) shows four examples representing different dynamical processes, shown in the order of SW transition, knockout, direct exchange, and unchanged structure. All of these beam-induced dynamics of P dopants are initiated by an out-of-plane momentum imparted on PKA by the backscattering of a single electron, which occurs stochastically with a small probability. The definitions of spherical coordinates θ and φ (momentum direction of the PKA whose energy is *E*) are plotted in the first frame of Fig. 2G, along with an example of an unchanged structure (θ = 25°, φ = 285°, with the kinetic energy *E* = 15.0 eV). If the initial momentum is not strictly upward, but tilted at an angle (θ = 20°, φ = 75°, *E* = 15 eV in this case), then a SW transition occurs (Fig. 2D) (*25*). As an example of knockout in Fig. 2E, the initial momentum of PKA is tilted toward θ = 20°, φ = 180°, with *E* increased to 17.0 eV. Last, in Fig. 2F, an initial PKA momentum perpendicular to the plane (θ = 0°) yields a direct exchange when *E* = 17 eV.

From these plots, several conclusions can be drawn for the phosphorus dopant: (i) A SW transition can be initiated with a lower PKA energy *E* (starting from 15 eV) than direct exchange. (ii) Increasing from 15 to 17 eV, direct exchange gradually becomes the dominant dynamical process. (iii) When *E* reaches around 17 eV, knockouts begin to occur. (iv) Somewhat counterintuitively, direct exchange is easier when the PKA momentum is pointing away from the target P atom (φ = 180°), instead of pointing toward it (φ = 0°). As we shall see, these polar plot features are derivable from the PKS theory, from which the relative scattering cross sections of each configurational outcome can be estimated.

The replacement dynamics (Fig. 1D) are due to the freely diffusing C adatoms on graphene surface. In Fig. 2H, our calculation shows that C adatoms can bond stably on a C─C bridge close to the underside of a P site (shown as the initial state). By performing a cNEB calculation, we see that, to transit from this initial state to a final state where the P has been replaced by C, the system only needs to cross a 0.4-eV barrier, available thermally or from the 60-keV electron beam (*28*), and subsequently reducing the potential energy of the system by 4.5 eV.

**Fig. 3:**
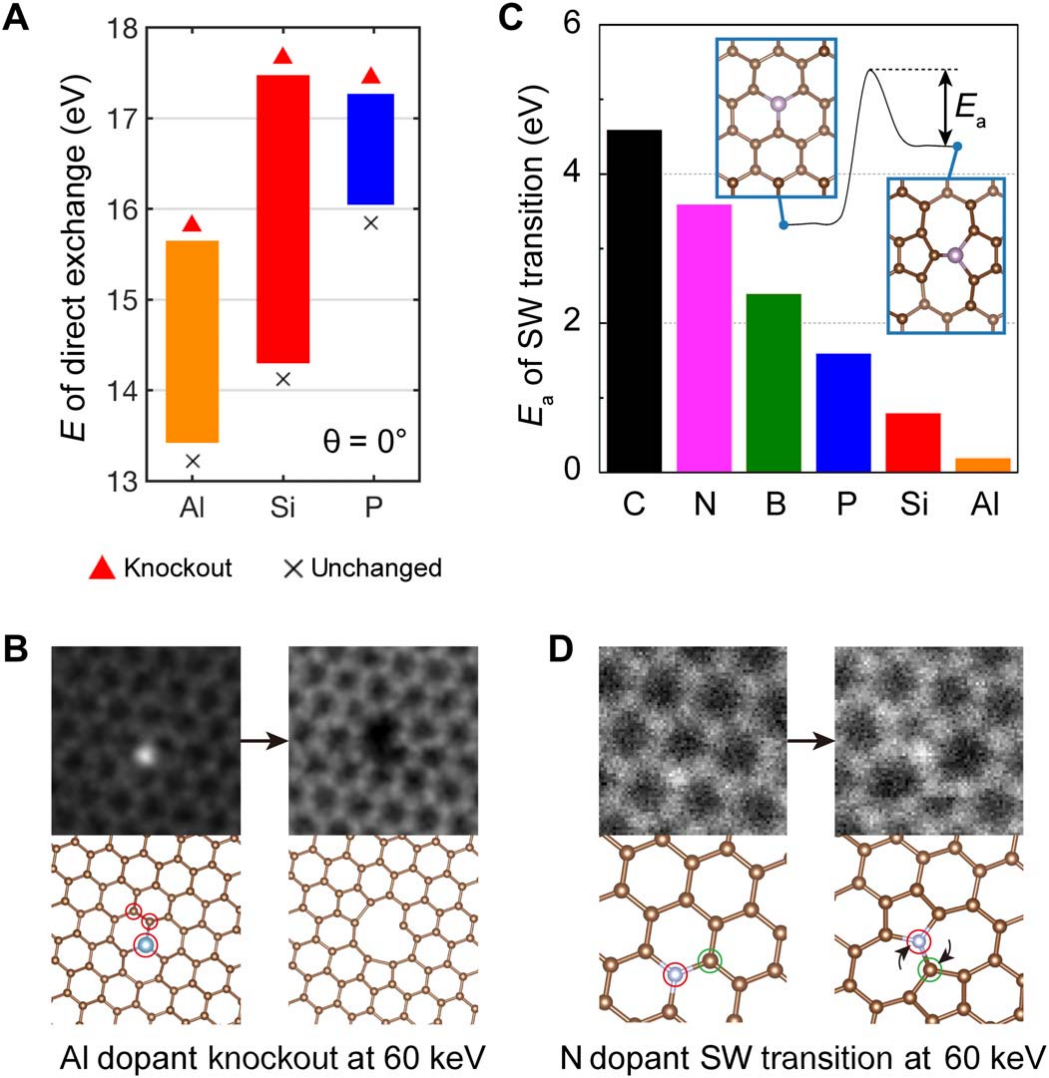
Comparison of dynamics of different impurity elements. (**A**) Comparison of the direct exchange energy ranges between Al, Si, and P for head-on collision (θ = 0°). (**B**) Experimentally, the knockout of an Al dopant and two carbon atoms nearby was observed after 7 min of continuous radiation at 60 keV, corresponding to the low displacement threshold predicted in (A). Red circles mark atoms displaced in the second frame. (**C**) The energy barriers (*E*_a_) of configurational change from 55-77 structures back to the pristine lattice are illustrated for various elements (C, 4.6 eV; N, 3.6 eV; B, 2.4 eV; P, 1.6 eV; Si, 0.8 eV; Al, 0.2 eV). Inset: The definition of *E*_a_ in the energy profile of the SW transition, where the original curves can be found in fig. S4. (**D**) An experimentally observed SW transition of an N dopant at 60 keV.

Comparing different graphene dopants, we found that P hops less actively in experiment than what has been reported for Si (*22*). To explain this, we compare the PKS-predicted energy range of direct exchange for Si, P, and Al cases when assuming a head-on collision (θ = 0°; Fig. 3A). We find that the Si case covers the greatest energy range, resulting in larger probability of direct exchange than for P. The displacement threshold of the C neighbor of an Al dopant is much lower than those for Si and P, so knockout of the PKA is a more likely outcome for Al dopants. We have observed an Al dopant and its surrounding atoms being knocked out together by a 60-keV electron beam (Fig. 3B), while we never observe this process for Si or P at the same electron energy. This implies that a lower acceleration voltage (${\tilde{E}}_{e}$) could help facilitate direct exchange also for Al.

On the contrary, a SW transition is more likely to be observed for a P dopant compared with Si. Related cNEB calculations are shown in Fig. 3C. As a broader comparison, we compute six elements, including C, N, B, P, Si, and Al, any of which theoretically could experience a SW transition. To be able to observe the SW transition in STEM, the 55-77 structure must be sufficiently stable to capture an image frame. Its stability is proportional to the depth of the potential energy well of the 55-77 structure (energy barrier between the highest energy transition state and the 55-77 structure), which is given as the activation energy *E*_a_ (the original cNEB curves can be found in fig. S4). We note that the cNEB calculation can only provide qualitative ranking, not quantitative characterization of the beam-induced dynamical process, since the electron-imparted momentum is localized on the PKA and does not necessarily exactly follow the collective reaction pathway of the minimum energy path. The stability of 55-77 structures follows the order C > N > B > P > Si > Al. Among all the dopants we observed, we indeed find that N has the most stable 55-77 structure [Fig. 3D; the single-atom electron energy-loss spectroscopy (EELS) characterization of this particular N dopant can be found in (*29*)]. Purely thermally, for a preexponential factor of 2 × 10^12^/s estimated from harmonic transition-state theory analysis in (*30*), the Si 55-77 structure back-transformation rate at 300 K is 0.073/s, making such defects (and all the dopants with a higher energy barrier), in principle, STEM observable if they are created.

### PKS formalism

**Fig. 4:**
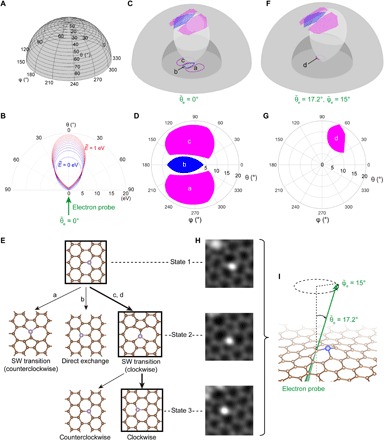
PKS: A scheme for evaluating cross sections of different dynamic processes. (**A**) The spherical coordinate system used for describing the PKS (with θ and φ defining the direction of momentum, and the radius defining the postcollisional kinetic energy, *E*, of the C neighbor). (**B**) A vertical cross section of the PKS showing the distribution of function *f* (dubbed “ovoid” hereafter) for the upward 60-keV electron beam (${\tilde{\theta }}_{e}=0^{{}^{\circ}}$) interacting with a moving PKA ($\tilde{E}=0$ to 1 eV). (**C**) The ovoid of a vibrational PKA (we use $\tilde{E}=0.5$ eV here for the amplified illustration) intersects with different outcome areas, where in (**D**), the intersections are projected to a polar plot. The magenta areas marked with a and c represent SW transitions (clockwise and counterclockwise, respectively), and the blue area marked with b represents direct exchange. (**E**) A decision tree showing possible outcomes of the atom-electron interaction, where the probability of going through each path is proportional to the cross sections. (**F**) The PKS and the ovoid of a tilted electron beam (${\tilde{\theta }}_{e}=17.2^{{}^{\circ}},{\tilde{\varphi }}_{e}=15^{{}^{\circ}}$) acting on a vibrational PKA ($\tilde{E}=0.5$ eV), with (**G**) showing a different intersection projected to the polar plot. Here, only clockwise SW transitions are activated, marked with d in the magenta area. (**H**) An experimentally observed clockwise SW transition of a Si dopant activated in a tilted sample as in (F) and (G). Three corresponding stages are placed alongside the decision tree in (E), where the experimental states are marked by black squares, and the observed path is indicated by the thicker branches. Field of view: 1 nm × 1 nm. (**I**) A side perspective view of the electron beam tilted with respect to the graphene plane. The sample was kept tilted like this throughout all the frames in (H).

Predicting and comparing the scattering cross sections of different dynamic processes within a unified framework is essential for atomic engineering, so we have developed the PKS formalism. Illustrated on the polar plots in Fig. 2 (A to C), the azimuthal angle φ and polar angle θ correspond to the direction of the momentum of postcollisional PKA (Fig. 4A), and the radius of the polar plot represents its kinetic energy *E* (Fig. 4B). Every point in PKS describes the momentum status of the PKA in terms of its momentum direction and kinetic energy right after collision (*t* = τ_c_), all of which lead to a dynamic outcome that correspond to the points in Fig. 2 (A to C). In Fig. 4C, these outcomes are grouped to differently colored blocks represented in three dimensions in PKS. The momentum distribution of PKA after an electron collision has an ovoid profile, whose shape changes with respect to the energy and direction of an incoming electron and the precollisional momentum of the atom. We conceptualize this momentum distribution as a “Doppler amplification effect” because small changes in precollisional momentum can lead to a much greater change of the postcollision momentum, as illustrated in Fig. 4B. While the theory presented in Materials and Methods is more general, for the illustration here, only atoms vibrating perpendicular to the graphene plane are considered. The Doppler amplification effect is essential here because our calculation shows that, if there were no precollisional kinetic energy of the atom, then there would not be a chance of direct exchange, SW transition, or knockout of a carbon neighbor in the experiments (Fig. 1). In Fig. 4C, the intersection of the colored regions and the ovoid of a vibrating carbon atom (we use $\tilde{E}=0.5$ eV here for the amplified illustration) interacting with a 60-keV electron is projected to the polar plot in Fig. 4D, where areas a, b, and c correspond to regions of counterclockwise SW transition, direct exchange, and clockwise SW transition. The existence of these three intersections implies that all of the above dynamic processes are possible when the electron beam is pointing strictly upward (${\tilde{\theta }}_{e}$ = 0°), due to the possibility of the electron scattering to an angle and transferring some lateral momentum (*24*, *31*). A detailed formulation of the PKS theory can be found in Materials and Methods.

For atomic engineering, we use a decision tree to show the possible paths of evolution (Fig. 4E), with its root node indicating the initial structure, and the child nodes indicating the next possible structures with different branching probabilities. In this example, the root trifurcates into three different paths, corresponding to the three different dynamic processes. By using the PKS formalism and taking the mean squared velocity of the carbon atom to be 3.17 × 10^5^ m^2^ s^−2^ (*32*) or $\tilde{E}=0.02$ eV, we can calculate the relative probability between the SW exchange and direct exchange as ~63.4. Experimentally, we find this number to be somewhat higher at ~224 (11.2/0.05 barn, as shown in Table 1). Apart from the approximation we have done in our calculation, abMD overestimates the energy required for inducing dynamics by about 10% (*5*, *33*), partly due to possible inaccuracies in the description of bond breaking in standard (semi-) local DFT (*34*), and the scheme we have constructed here does not consider electronic excitations. For these reasons, we considered only relative probabilities in the PKS above. Further improvements in theoretical modeling are needed before quantitative predictions are possible for impurity sites in graphene (*5*) and to account for the in-plane vibration components of the C atoms. However, we want to stress here that PKS correctly predicts that the SW transition has a much higher probability than direct exchange, whereas static in-plane transition paths calculated with cNEB cannot rationalize the branching ratio of the two processes.

**Table 1:** Experimentally estimated cross sections for each dynamic process under the 60 keV electron irradiation. The geometric mean provides a good estimate for the underlying Poisson expectation value (*19*). For comparison, typical elastic scattering cross sections for carbon are ~10^5^ to 10^6^ barn for high-angle annular dark-field (HAADF), while inelastic K-edge ionization is ~10^4^ barn (both in EELS and energy-dispersive x-ray), and plasmon generation is ~10^6^ barn (*41*). In graphene, the ratio of plasmon and core loss ionization cross sections can be much higher, up to 10^4^ (*42*). The SW transition of P dopant from 55-77 to hexagonal is too fast to resolve with our imaging speed, so a lower limit is provided.

**Dopants**	**Dynamics**	**Cross sections (barn, geometric mean ± SE)**
P	Direct exchange	0.05 ± 0.02
	SW transition (hexagonal to 55-77)	~11.2
	SW transition (55-77 to hexagonal)	> 200
	Replacement (by freely diffusing C adatoms)	0.07 ± 0.02
	Knockout (of PKA, the C neighbor)	0.01 ± 0.003
N	SW transition (hexagonal to 55-77)	0.9 ± 0.1
	SW transition (55-77 to hexagonal)	22.0 ± 4.5
Al	Knockout (of PKA, the C neighbor)	~0.25

To further experimentally test our theory, we tilted a Si-doped sample so that the electron beam was incident at a specific angle (${\tilde{\theta }}_{e}$= 17.2°, ${\tilde{\varphi }}_{e}$ = 15°, determined from the calibrated double-tilt sample holder; because of their similar covalent size and bonding, we expect the relative positions of outcome functions of threefold Si and P impurities to be similar). Based on our calculations, with such a tilt, the direct exchange and the counterclockwise SW transition will be totally suppressed, leaving only clockwise SW transition active (Fig. 4, F and G). In this proof-of-principle experiment, we indeed observed only clockwise SW transitions (Fig. 4H), demonstrating control of this configurational outcome. Thereafter, from the 55-77 structure back to the honeycomb lattice, clockwise SW transition is again the only active dynamic process (fig. S9).

## DISCUSSION

The long-term vision of atomic engineering is to precisely position individual atoms with desired internal states, including the nuclear spin, image and control atomic assemblies from 1 to 1000 atoms, and use precisely controlled atoms and their electronic/nuclear states for devices such as atomic clock and memory. Successful atomic engineering comes from understanding two parts: (i) how the desirable local configurational changes can be induced to increase the speed and success rate of control and (ii) how to scale up these basic unit processes into feasible structural assemblies of 1 to 1000 atoms to produce the desired functionality. Here, we focused on the first part by surveying the single-step dynamics of graphene dopants, primarily phosphorus, caused by electron irradiation both in experiment and simulation, and developed a theoretical scheme for describing the probabilities of competing configurational outcomes through the postcollisional momentum vector of the PKA. However, a brief description of the second part is also warranted.

What one would want is to arrive at a predesigned configurational state *i*_final_ ≡ {***r****_n_*} of the atoms as quickly as possible, through a series of collisions with focused electrons, which are known to have enough energy to displace atoms in the radiation damage context (*35*) but exploited here in a controlled fashion to bias the configurational evolution, some of which may conserve mass locally and some of which may not. We start with an initial configurational state *i*_initial_ that is precisely imaged and wish to travel across intermediate configurations … → *i* → *i*′ → *i″* → v… and finally arrive at *i*_final_, similar to playing Rubik’s cube but with probabilities. One obviously must balance “risk” against “speed” in playing this game, since there could exist trap states {*i*_trap_} that severely delay the arrival to *i*_final_ or even make achieving *i*_final_ impossible (for example, fourfold-coordinated P is a trap state with a 60-keV e-beam, since we found that it is no longer possible to further alter the configuration once it is reached). Through the PKS formalism, we see that ideally we can affect *i* through the following control variables: (a) choosing the PKA atom and the e-beam spot center, (b) choosing the FWHM of the e-beam and the beam dose, (c) choosing ${\tilde{E}}_{e}$, and (d) choosing (${\tilde{\theta }}_{e},{\tilde{\varphi }}_{e}$) by tilting the sample or the beam, with the constraint that one must also be able to simultaneously image the sample for feedback control. The probabilistic nature of this tree traversal game makes it similar also to playing soccer. Computational prediction of the branching ratios and the absolute transition rate, even if approximate, would be the key for any kind of engineering optimization of the total risk/speed tradeoff. Conceivably, one could apply machine learning (*36*) and artificial intelligence to understanding the unit processes, as well as the assembly process, in the future. However, first-principles theory has, at this stage, been demonstrated to be tremendously helpful.

Specifically, here, we have categorized four types of electron-induced dynamics of atomic dopants on graphene and constructed a scheme for controlling them. By explaining the mechanisms for each process by first-principles calculations, we provide a convenient categorization for generic dopant dynamics. We have demonstrated the possibility of electron-beam manipulation of P and selectively induced directional SW transitions of Si. A vector-space theory (PKS) is proposed for calculating the relative ratio of scattering cross sections between different configurational outcomes, corresponding to branching probabilities in a decision tree. The two main ingredients of this theory, the outcome functions and the momentum-resolved differential cross sections (see Materials and Methods), are assessed and numerically computed by abMD and analytical relativistic electron collision kinematics, respectively. A “Doppler amplification effect” is discussed whereby a small change in precollisional PKA momentum results in a larger change in PKS momentum due to momentum conservation.

The PKS theory is developed on the basis of the fortunate separations of time scales of relativistic electron collision (τ_c_ ~ 10^−22^ s), electronic excitation (a penetration event, τ_e_ ~ 10^−18^ s), collective postpenetration electronic relaxation (τ_E_ ~ 10^−15^ s), ionic trajectory (τ_I_ ~ 10^−12^ s) leading to configurational change, and the frequency of these penetrations (τ_p_ ~ 10^−9^ s). While momenta will eventually be dephased after τ_I_, what the PKA momentum vector was and how this vector may evolve up to τ_I_ are essential, so this is a truly dynamical problem. Up to τ_c_, we have a relativistic collision problem that is PKA nucleus dependent but crystal independent. The physics revealed and the computational/analytical framework developed in this paper are general and can further help develop techniques for controlling single-atom dynamics in 3D materials (*23*), and ultimately, upscaling manipulations of multiple atoms to assemble 1 to 1000 atoms with high speed and efficacy.

## MATERIALS AND METHODS

### Chemical vapor deposition

Phosphorus-doped graphene was synthesized using chemical vapor deposition. First, a 25-μm-thick Cu foil (Alfa Aesar, no. 13382) was washed in 5% HCl solution for 3 min and then rinsed in deionized water several times. After that, the Cu foil was dried by nitrogen and quickly loaded into a tube reactor (diameter, 1 inch; length, 1.5 m). A quartz boat container with about 100 mg of triphenylphosphine (C_18_H_15_P; Sigma-Aldrich) used as a sole precursor source was placed upstream from the sample. The system was evacuated to a vacuum lower than 1 × 10^−3^ Pa. The zone-1 of the furnace was first heated to 1050°C at a rate of 20°C/min in 25 and 100 sccm of H_2_ and Ar, respectively. After annealing for 20 min, the temperature was decreased to 1000°C. Then, zone-2 of the furnace was heated to 80°C at a rate of 5°C/min. The triphenylphosphine vapor was carried into zone-1 by the flowing H_2_ and Ar, initiating graphene growth on the Cu foil. After 20 min, the system was cooled to room temperature with a cooling rate of about 50°C/min by opening the furnace. During growth and cooling, the flux of H_2_ and Ar remained unchanged. The resulting P-doped graphene was then transferred onto Quantifoil Au TEM grids for electron microscope imaging.

### Electron microscopy

The atomic structure of the sample was observed using the aberration-corrected Nion UltraSTEM 100 at the Oak Ridge National Laboratory’s Center for Nanophase Materials Sciences User Facility operated at 60 kV, and the same instrument at the University of Vienna. In a standard treatment, the sample was baked in vacuum at 160°C for 8 hours before insertion into the microscope column. The vacuum level at the sample volume during the experiments was <3 × 10^−9^ mbar. The convergence semiangle of electron probe is 30 mrad, and the collection semiangle of the electron energy loss spectrum is 48 mrad. The medium angle annular dark field (MAADF) collection semiangle range is 54 to 200 mrad. Electron current was kept in between 50 and 60 pA during all of the imaging. To estimate the doses of the spot irradiation manipulation experiments, we used a model of the expected probe intensity profile as described in (*2*, *22*).

### Postprocessing of images

The postprocessing of images was done to enhance their contrast and clarity. Figures 1 (A and B) and 3 (B and D) were processed in ImageJ using a median filter with a kernel radius of 2 pixels. Figure 1 (C and D) was binned by 0.5 (ImageJ-Scale) and a slight Gaussian blur (radius = 0.5), whereas Fig. 1 (E and F) are raw figures averaged by aligning and stacking several frames on top of each other. The insets of Figs. 1 (E and F) and 4H were processed using a double Gaussian filter plugin in ImageJ with parameters sigma1 = 0.21, sigma2 = 0.15, and weight = 0.3 (*12*) and then colored using lookup table “cyan.” Apart from Fig. 1 (E and F), all other dynamics were recorded during scanning.

### First-principles calculations

abMD was performed using DFT within the generalized gradient approximation in the form of Perdew-Burke-Ernzerhof’s exchange-correlation functional (*37*). The time step was chosen to be 1 fs, after testing that this is sufficient for predicting the dynamics (we simulated the direct exchange of P using a time step of 0.1 fs, but found no difference within the precision of our calculation ±0.1 eV). All calculations were performed using the Vienna Ab Initio Simulation Package (*38*), and the plane-wave cutoff energy was chosen to be 300 eV with PREC set to Normal?.

### PKS formalism

Every point in PKS describes the momentum status of the PKA in terms of its momentum direction and kinetic energy immediately after collision, which can be identified by a triplet Γ ≡ (θ, φ, *E*). Similarly, the energy-momentum triplet of a precollision electron (*t* = 0^−^) will be denoted by ${\tilde{\Gamma }}_{e}\equiv ({\tilde{\theta }}_{e},{\tilde{\varphi }}_{e},{\tilde{E}}_{e})$ and that of a precollision PKA (*t* = 0^−^) will be denoted by $\tilde{\Gamma }\equiv (\tilde{\theta },\tilde{\varphi },\tilde{E})$. The PKS differential volume is denoted by *d*Γ = *E*^2^*d*Ω*dE*, where *d*Ω is the solid angle of the postcollisional PKA momentum direction and has a unit of eV^3^ despite conveying momentum vector-space information (one can think of Γ-space as a transformed momentum space with easy-to-read labels in electron volts).

The PKS framework involves a two-step process: (i) electron scattering from the nuclear potential of the PKA, denoted by “electron → PKA” (a 0.1 zeptosecond–time scale interaction, τ_c_ ~10^−22^ s) and described by function *Q*, the PKA momentum resolved electron differential scattering cross section; (ii) the ensuing dynamics of the PKA, denoted by “PKA → configurational change” (a picosecond–time scale interaction, τ_I_ ~10^−12^ s) described by *P_i_*, the probability that outcome *i* will take place. For every energy-momentum triplet Γ in PKS, the outcome functions *P_i_*(Γ) describe the probability that such a scattering event leads to an outcome configuration of unchanged (*i* = 0), direct exchange (*i* = 1), SW transition (*i* = 2), knockout (*i* = 3), etc., which is crystal structure dependent, and with 0 ≤ *P_i_*(Γ) ≤ 1, ∑*_i_P_i_*(Γ) = 1. Thermal and quantum perturbations of the surrounding crystal structure can smear the branching rates and make *P_i_*(Γ) neither 1 nor 0, but because *E* has a much larger magnitude than such surrounding fluctuations, there tends to be a dominant outcome *c*(Γ) ≡ arg max*_i_ P_i_*(Γ) for every Γ (*c* stands for “configurational outcome” denoted by different colors). For example, if direct exchange is the most probable outcome at Γ, then *c*(Γ) = 1; if SW transition dominates at Γ, then *c*(Γ) = 2, etc. We used *c*(Γ) to partition the PKS into different color blocks in the 3D visualization scheme shown in Fig. 4C (we use the color blue for *i* = 1, magenta for *i* = 2, etc.). In addition, *c*(Γ) = 0 for regions where recovering to the same configuration is the dominant outcome. Different total cross sections of dynamic processes can be calculated considering the following two consecutive processes.

### “Electron → PKA” process

We introduce an intermediate function $Q(\Gamma ;{\tilde{\Gamma }}_{e})$, which has units of barn/eV^3^, to describe the probability that a single penetrating electron can eject the PKA into a particular differential PKS volume dΓ (units of eV^3^) by impinging on the corresponding impact-parameter differential area *d*σ = $Q(\Gamma ;{\tilde{\Gamma }}_{e})d\Gamma$. *Q* is essentially a probability density distribution, partly due to the impact-parameter dependence of the electron-PKA collision and the probabilistic nature of $\tilde{\Gamma }$, the precollision PKA momentum, which has been shown to be important (*32*, *39*). *Q* can be computed as

$$Q(\Gamma ;{\tilde{\Gamma }}_{e})=\int d^{3}\tilde{\Gamma }\times \tilde{P}(\tilde{\Gamma })\times q(\Gamma ,\tilde{\Gamma };{\tilde{\Gamma }}_{e})$$ (1)

where $\tilde{P}(\tilde{\Gamma })$ is the probability distribution of PKA momentum before collision (*t* = 0^−^) (*32*, *40*) and $d^{3}\tilde{\Gamma }\equiv {\tilde{E}}^{2}\text{sin}\;\tilde{\theta }d\tilde{E}d\tilde{\theta }d\tilde{\varphi }$ is its differential volume. The function $q(\Gamma ,\tilde{\Gamma };{\tilde{\Gamma }}_{e})$ describes the energy-momentum–resolved cross section of PKA parameterized by $\tilde{\Gamma }$

$$q(\Gamma ,\tilde{\Gamma };{\tilde{\Gamma }}_{e})\equiv \frac{1}{E^{2}}\frac{d^{2}\sigma }{d\Omega \mathrm{dE}}=\frac{1}{E^{2}}\frac{d\sigma }{d\Omega }(\Gamma ,\tilde{\Gamma };{\tilde{\Gamma }}_{e})\times \delta [E-f(\theta ,\varphi ,\tilde{\Gamma };{\tilde{\Gamma }}_{e})]$$ (2)

where $\frac{d\sigma }{d\Omega }(\Gamma ,\tilde{\Gamma };{\tilde{\Gamma }}_{e})$ is the angular-resolved differential cross section of electron-atom scattering derived from McKinley-Feshbach formalism (*33*, *34*), which describes the scattering probabilities of PKA with respect to its outgoing angles and energy. The δ function in energy is the result of energy-momentum conservation and is independent of the details of the nuclear potential. The function *f* defines the energy contour of PKA with respect to the outgoing angles θ, φ given the status of incident electron ${\tilde{\Gamma }}_{e}$, and precollision PKA, $\tilde{\Gamma }$. We used a relativistic treatment to obtain *f* as shown in eq. S10 in the Supplementary Materials. The $q(\Gamma ,\tilde{\Gamma };{\tilde{\Gamma }}_{e})$ function, parametrized by the incident electron energy ${\tilde{E}}_{e}$ and momentum direction ${\tilde{\theta }}_{e}$, ${\tilde{\varphi }}_{e}$, describes the scattering from the nuclear potential and thus does not depend on the crystal structure.

### “PKA → configurational change” process

The total cross section of a dynamic process *i* can then be computed by integrating *Q* in Eq. 1 weighted by the outcome function *P_i_* over the whole PKS

$$\sigma_{i}({\tilde{\Gamma }}_{e})=\int d^{3}\Gamma \times P_{i}(\Gamma )\times Q(\Gamma ;{\tilde{\Gamma }}_{e})$$ (3)

where *d*^3^Γ ≡ *E*^2^ sin θ*dEd*θ*d*φ is the PKS differential volume element for postcollisional PKA. The cross sections of different dynamic processes are functions of ${\tilde{\Gamma }}_{e}$, indicating that the probabilities of different dynamics can be tuned by the energy of electron $\left({\tilde{E}}_{e}\right)$ or by the incident angles $\left({\tilde{\varphi }}_{e},{\tilde{\theta }}_{e}\right)$ with respect to the sample, which can be tuned by tilting the beam or the sample. These are the primary control variables of atomic engineering, along with the selection of the PKA, and the electron beam profile which overlaps with the barn-scale areas centered on this PKA.

In computer-controlled atomic engineering, in evaluating Eq. 3, although $Q(\Gamma ;{\tilde{\Gamma }}_{e})$ has many dependent variables and Eqs. 1 and 2 look complicated, they are analytical integrals and thus can be evaluated on the fly. *P_i_*(Γ), however, is crystal and material dependent, and needs to be precomputed with expensive ab initio calculations, and tabulated or machine learned (*36*) for efficient evaluation of Eq. 3.

For simplicity, in the graphical illustrations in the main text, the “PKA → configurational change” dynamics are assumed to be deterministic, making *P_i_*(Γ) either 0 or 1, without any smearing at the boundaries. This is reflected in Fig. 4C as the sharp boundaries of the PKS regions, where the probability of configurational outcome *i* is 1 within the boundary and is 0 everywhere else. On the other hand, the contour of $\delta [E-f(\theta ,\varphi ,\tilde{\Gamma };{\tilde{\Gamma }}_{e})]$ is an ovoid with infinitely thin shell in PKS. The electron cross section of certain configurational outcome, σ*_i_*, can thus be visualized easily in this fictitious limit of no thermal or quantum uncertainties: The intersection areas between the ovoid and the *c*(Γ) = *i* regions represent the part of PKS space that can induce certain configurational change *i*, which is then integrated with *d*σ/*d*Ω to get the total cross section for each of them.

To complicate the picture slightly, however, for a quantitative description of the outcomes, it has been shown that the precollisional momentum $\tilde{\Gamma }$ of the PKA is significant and important (*32*, *39*), due to what we may conceptualize as a “Doppler amplification effect” on Γ. To illustrate this with an approximate example (see section S6 for details), the outgoing velocity, v, of a PKA with precollisional vibrational velocity, $\tilde{v}$, can be well approximated by $v\approx v_{0}+\tilde{v}$, where $v_{0}$ is the postcollisional velocity of a static PKA. Squaring the two sides yields the energy equation $E\approx E_{0}+Mv_{0}\cdot \tilde{v}+\tilde{E}$. A small change in $\tilde{E}$ may result in up to ~10× change in *E* due to the second term $Mv_{0}\cdot \tilde{v}$, since $v_{0}$ is significantly larger than $\tilde{v}$ (because $v_{0}$ corresponds to energy of 10 eV, whereas $\tilde{v}$ corresponds to energy of ~0.1 eV). Therefore, a change as small as 0.1 eV due to thermal and quantum zero-point fluctuations in the precollision nuclear kinetic energy can change the PKA postcollision kinetic energy by as much as 1 eV, which subsequently can significantly alter the outcome probabilities. In momentum space, it is shown that the in-plane vibration also contributes to the amplification effect (see section S6). This necessitates a careful integral treatment in Eq. 1, where the infinite thin-shelled differential cross section $q(\Gamma ,\tilde{\Gamma };{\tilde{\Gamma }}_{e})$ will be smeared into a bowling pin–shaped probability density $Q(\Gamma ;{\tilde{\Gamma }}_{e})$ that depends on the precollisional velocity distribution (fig. S7). Postcollision, after a short period of τ_E_, the PKS momentum distribution $Q(\Gamma ;{\tilde{\Gamma }}_{e})$ will be convoluted with *P_i_*(Γ), a crystal-dependent quantity that one can precompute with abMD that integrates the evolution of atom trajectories on the ground-state BO surface (since we are beyond τ_E_). The overlap of $Q(\Gamma ;{\tilde{\Gamma }}_{e})$ (nuclear collisional kinematics) and *P_i_*(Γ) (crystal structure–dependent transition probability) in PKS space gives the net rate of configuational change (→*i*), after which the correlated atomic momenta dephase and the momenta correlation information is lost, leaving only heat (which subsequently conducts away or radiates out with yet another, longer timescale). All these happen (or not) long before the next electron penetrates the system, and the system configuration evolves (*i* → *i*′ → *i*″ → …) without carrying the detailed phase information about atomic momenta, so an uncorrelated probability distribution function of PKA momentum $\tilde{P}(\tilde{\Gamma })$ is all we need for characterizing this driven system for the next collision.

## Supplementary Material

http://advances.sciencemag.org/cgi/content/full/5/5/eaav2252/DC1

Download PDF

## SUPPLEMENTARY MATERIALS

Supplementary material for this article is available at http://advances.sciencemag.org/cgi/content/full/5/5/eaav2252/DC1 Section S1. Overview micrographs Section S2. Energy transfer from a 60-keV electron to a moving carbon atom Section S3. EELS characterization of P and Al dopants Section S4. Comparison of cNEB curves of various elements Section S5. Primary knock-on space Section S6. Ovoid modification by atom vibration (Doppler amplification effect) Section S7. Atomic engineering: Manipulation decision tree Section S8. Method of calculating experimental cross section Fig. S1. STEM image of P-doped graphene. Fig. S2. Energy transfer to vibrating carbon atom. Fig. S3. EELS of P and Al dopant. Fig. S4. cNEB curves. Fig. S5. Construction of PKS. Fig. S6. Selective dynamics by tilting beam. Fig. S7. Ovoid modification by vibration. Fig. S8. Decision tree for atomic engineering. Fig. S9. Selective dynamics from 55-77 structure back to honeycomb.
