# Supplementary material for: Engineering single-atom dynamics with electron irradiation
Source: Sci Adv. 2019 May 17;5(5):eaav2252. doi: 10.1126/sciadv.aav2252 (PMC6524980; doi:10.1126/sciadv.aav2252)
Supplement: http://advances.sciencemag.org/cgi/content/full/5/5/eaav2252/DC1 [file supp_5_5_eaav2252__index.html]

Science Advances | Science Advances

## Supplementary Materials

**This PDF file includes:**

- Section S1. Overview micrographs
- Section S2. Energy transfer from a 60-keV electron to a moving carbon atom
- Section S3. EELS characterization of P and Al dopants
- Section S4. Comparison of cNEB curves of various elements
- Section S5. Primary knock-on space
- Section S6. Ovoid modification by atom vibration (Doppler amplification effect)
- Section S7. Atomic engineering: Manipulation decision tree
- Section S8. Method of calculating experimental cross section
- Fig. S1. STEM image of P-doped graphene.
- Fig. S2. Energy transfer to vibrating carbon atom.
- Fig. S3. EELS of P and Al dopant.
- Fig. S4. cNEB curves.
- Fig. S5. Construction of PKS.
- Fig. S6. Selective dynamics by tilting beam.
- Fig. S7. Ovoid modification by vibration.
- Fig. S8. Decision tree for atomic engineering.
- Fig. S9. Selective dynamics from 55-77 structure back to honeycomb.

Download PDF

**Files in this Data Supplement:**

- Adobe PDF - aav2252\_SM.pdf
