# Supplementary material for: Engineering single-atom dynamics with electron irradiation
Source: Sci Adv. 2019 May 17;5(5):eaav2252. doi: 10.1126/sciadv.aav2252 (PMC6524980; doi:10.1126/sciadv.aav2252)
Supplement: Download PDF [file aav2252_SM.pdf]

Science Advances
AAAS

advances.sciencemag.org/cgi/content/full/5/5/eaav2252/DC1

Supplementary Materials for

Engineering single-atom dynamics with electron irradiation

Cong Su*, Mukesh Tripathi, Qing-Bo Yan, Zegao Wang, Zihan Zhang, Christoph Hofer, Haozhe Wang, Leonardo Basile, Gang Su, Mingdong Dong, Jannik C. Meyer, Jani Kotakoski, Jing Kong, Juan-Carlos Idrobo, Toma Susi*, Ju Li*

*Corresponding author. Email: liju@mit.edu (J.L.); toma.susi@univie.ac.at (T.S.); csu@mit.edu (C.S.)

Published 17 May 2019, *Sci. Adv.* **5**, eaav2252 (2019)
DOI: 10.1126/sciadv.aav2252

This PDF file includes:

Section S1. Overview micrographs
Section S2. Energy transfer from a 60-keV electron to a moving carbon atom
Section S3. EELS characterization of P and Al dopants
Section S4. Comparison of cNEB curves of various elements
Section S5. Primary knock-on space
Section S6. Ovoid modification by atom vibration (Doppler amplification effect)
Section S7. Atomic engineering: Manipulation decision tree
Section S8. Method of calculating experimental cross section
Fig. S1. STEM image of P-doped graphene.
Fig. S2. Energy transfer to vibrating carbon atom.
Fig. S3. EELS of P and Al dopant.
Fig. S4. cNEB curves.
Fig. S5. Construction of PKS.
Fig. S6. Selective dynamics by tilting beam.
Fig. S7. Ovoid modification by vibration.
Fig. S8. Decision tree for atomic engineering.
Fig. S9. Selective dynamics from 55-77 structure back to honeycomb.

Supplementary Materials

Section S1. Overview micrographs

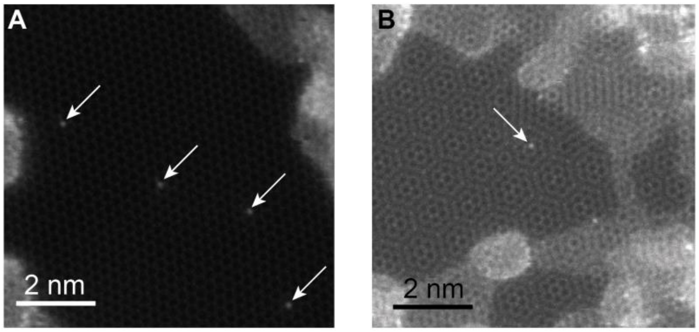

Fig. S1. STEM image of P-doped graphene. (A) Four three-fold coordinated P atoms within a clean area (approx. 10 nm in size) of a single-layer graphene sheet. **(B)** A three-fold coordinated P atom embedded in a clean double-layer graphene sheet with a twist angle of 15.3° (approx. 8 nm in size of clean area). All P atoms are marked by arrows.

Section S2. Energy transfer from a 60-keV electron to a moving carbon atom

The transferrable energy from a high energy electron to a static carbon atom can be estimated using the following relation

$$E = \frac{\tilde{E}_{\mathrm{e}}(\tilde{E}_{\mathrm{e}} + 1.02)}{496A}\sin^2\frac{\psi}{2} \quad \text{(S1)}$$

where $\tilde{E}_{\mathrm{e}}$ is the incident electron energy in MeV, E is the transferred energy to a *static* nucleus in MeV, *A* is atomic mass number (~12 for carbon), and y is the scattering angle of the electron. It can thus be estimated that the maximum energy transferable to a C atom is 10.9 eV for a 60 keV electron, which would mean that all dynamic processes should be prohibited since the lowest energy required for inducing a lattice change in P-doped graphene is larger than 14 eV as shown in main text.

However, the lattice of graphene is not static. Due to quantum mechanical zero-point and thermal vibrations, the atoms of the lattice are in motion, which relaxes the momentum conservation condition and increases the transferrable energy under the irradiation of a high energy electron beam. To simplify this picture, our following derivation assumes a head-on collision between an electron and a C atom ($q_{\mathrm{e}} = 0$), as shown in fig. S2.

Considering the conservation of momentum and energy of an electron-nucleus system, we have

$$\tilde{\vec{p}}_{\mathrm{e}} + \tilde{\vec{p}} = \vec{p}_{\mathrm{e}} + \vec{p} \tag{S2}$$

$$\tilde{E}_{\mathrm{e}} + \tilde{E} = E_{\mathrm{e}} + E \tag{S3}$$

where $\tilde{\vec{p}}_{\mathrm{e}}$, $\tilde{E}_{\mathrm{e}}$, $\vec{p}_{\mathrm{e}}$, and E_{e} denote the momentum and energy of *electron* before (with ˜) and after (without ˜) collision with $\tilde{\vec{p}}$, $\tilde{E}$, $\vec{p}$, and E being the counterparts for the *nucleus*. The momentum is related to energy relativistically for the electron, and non-relativistically for the nucleus

$$\left|\vec{p}_{\mathrm{e}}\right| = \frac{1}{c}\sqrt{2E_{\mathrm{e}}E_0 + E_{\mathrm{e}}^2} \tag{S4}$$

$$\left|\vec{p}\right| = \sqrt{2ME} \tag{S5}$$

and the pre-collision also follows the same form of the above equations. We therefore get

$$\frac{1}{c}\sqrt{2\tilde{E}_{\mathrm{e}}E_0 + \tilde{E}_{\mathrm{e}}^2} + \sqrt{2M\tilde{E}} = \sqrt{2ME} - \frac{1}{c}\sqrt{2(\tilde{E}_{\mathrm{e}} + \tilde{E} - E)E_0 + (\tilde{E}_{\mathrm{e}} + \tilde{E} - E)^2} \tag{S6}$$

Since the kinetic energy of the nucleus is much smaller than that of the electron in all stages, we can approximate $\tilde{E}_{\mathrm{e}} + \tilde{E} - E$ into $\tilde{E}_{\mathrm{e}}$, and get the following expression

$$\sqrt{2ME} = \sqrt{2M\tilde{E}} + \frac{2}{c}\sqrt{2\tilde{E}_{\mathrm{e}}E_0 + \tilde{E}_{\mathrm{e}}^2} \tag{S7}$$

For a static nucleus before collision ($\tilde{E} = 0$), we have

$$\sqrt{2ME_{\mathrm{stat}}} = \frac{2}{c}\sqrt{2\tilde{E}_{\mathrm{e}}E_0 + \tilde{E}_{\mathrm{e}}^2} \tag{S8}$$

and subtracting equation (S7) to (S8) gives

$$\sqrt{E} = \sqrt{\tilde{E}} + \sqrt{E_{\mathrm{stat}}} \tag{S9}$$

This relation between the final energy of PKA (E) and the atom vibration energy ($\tilde{E}$) can be plotted as fig. S2. Therefore, to activate a direct exchange process, a vibration energy of 0.5 eV is enough. Although the average kinetic energy of atoms at room temperature is much smaller

(0.025 eV), their velocities follow a normal distribution with a width given by the mean-square velocity (or corresponding kinetic energy), leading to a finite population of atoms with high kinetic energies at the moment of impact.

The probability distribution of out-of-plane velocities of carbon atoms in graphene can be estimated using equation (19) in Ref. 24. To get a sense of how rare these events are, the probabilities of vibrational energy above a certain level are: (1) 0.1 eV: 0.0122; (2) 0.2 eV: 7.2×10^{-4}; (3) 0.3 eV: 4.8×10^{-5}; (4) 0.4 eV: 3.3×10^{-6}; (5) 0.5 eV: 2.4×10^{-7}. Although large kinetic energies are rare, such vibrations do yield finite cross sections for events that would be otherwise forbidden.

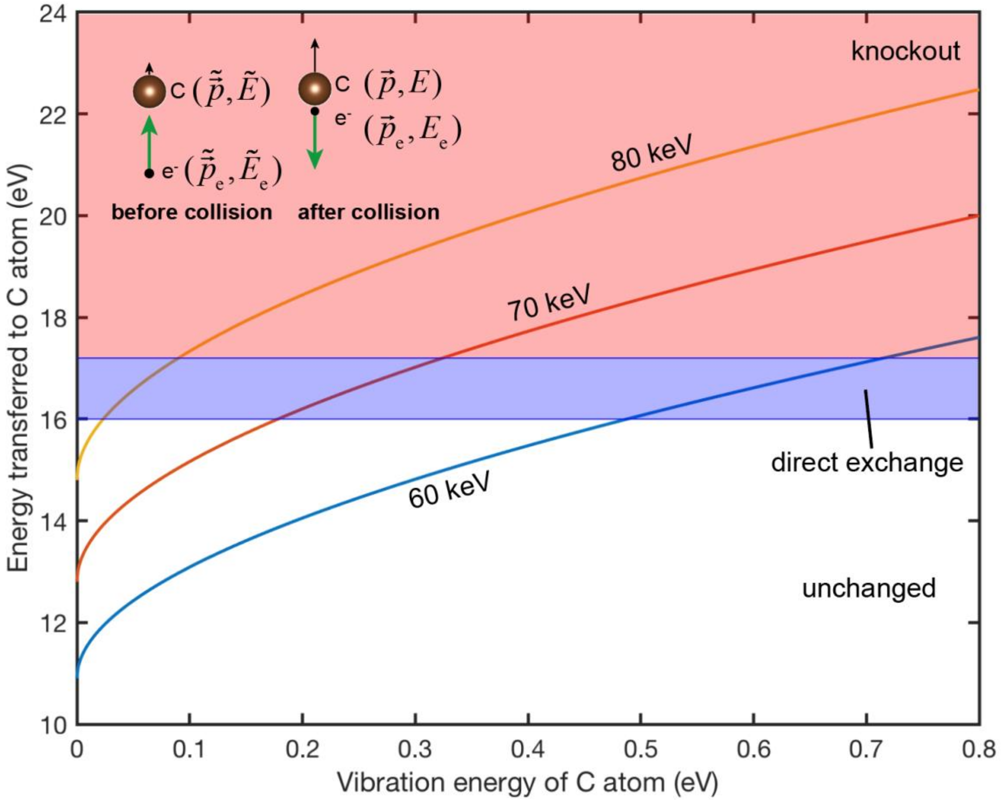

Fig. S2. Energy transfer to vibrating carbon atom. The red and blue shaded areas mark the direct exchange and knock-out zones for a C neighbor to P. Inset: Schematic illustration of head-on collision between electron and C atom, with annotations matching the derivation above.

Section S3. EELS characterization of P and Al dopants

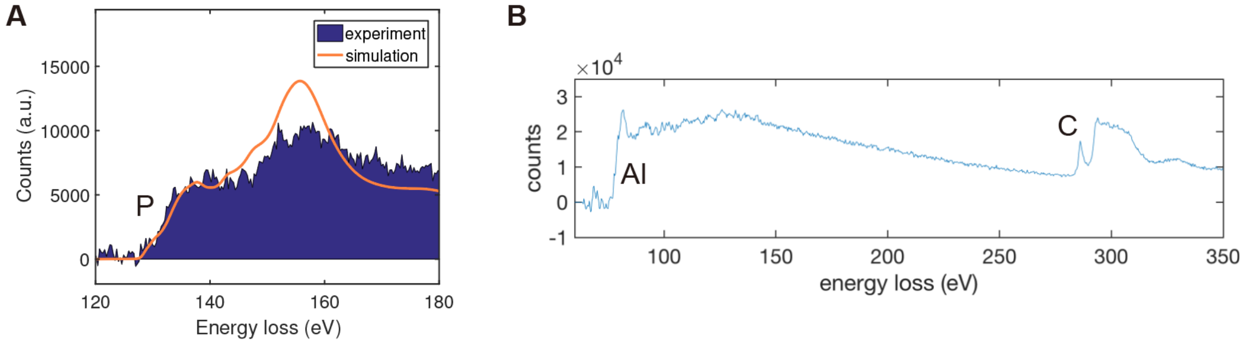

Fig. S3. EELS of P and Al dopant. (A) EELS acquired in experiment and overlaid with a multiple-scattering simulation (with a core-hole approximation) of a three-coordinated P dopant. **(B)** EELS showing the core loss edges of Al and C. All of the dopants shown in the main text have been characterized by EELS, and all EELS data has been calibrated to the C edge.

Section S4. Comparison of cNEB curves of various elements

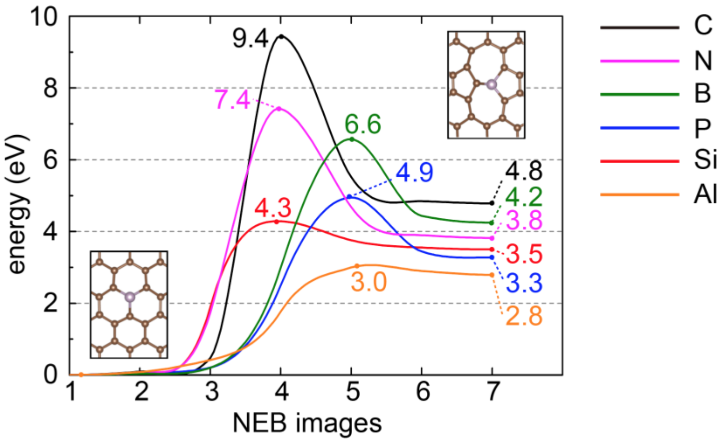

Fig. S4. cNEB curves. The E_a used in the main text is obtained from subtracting the final energy from the peak energy indicated in the plot.

Section S5. Primary knock-on space

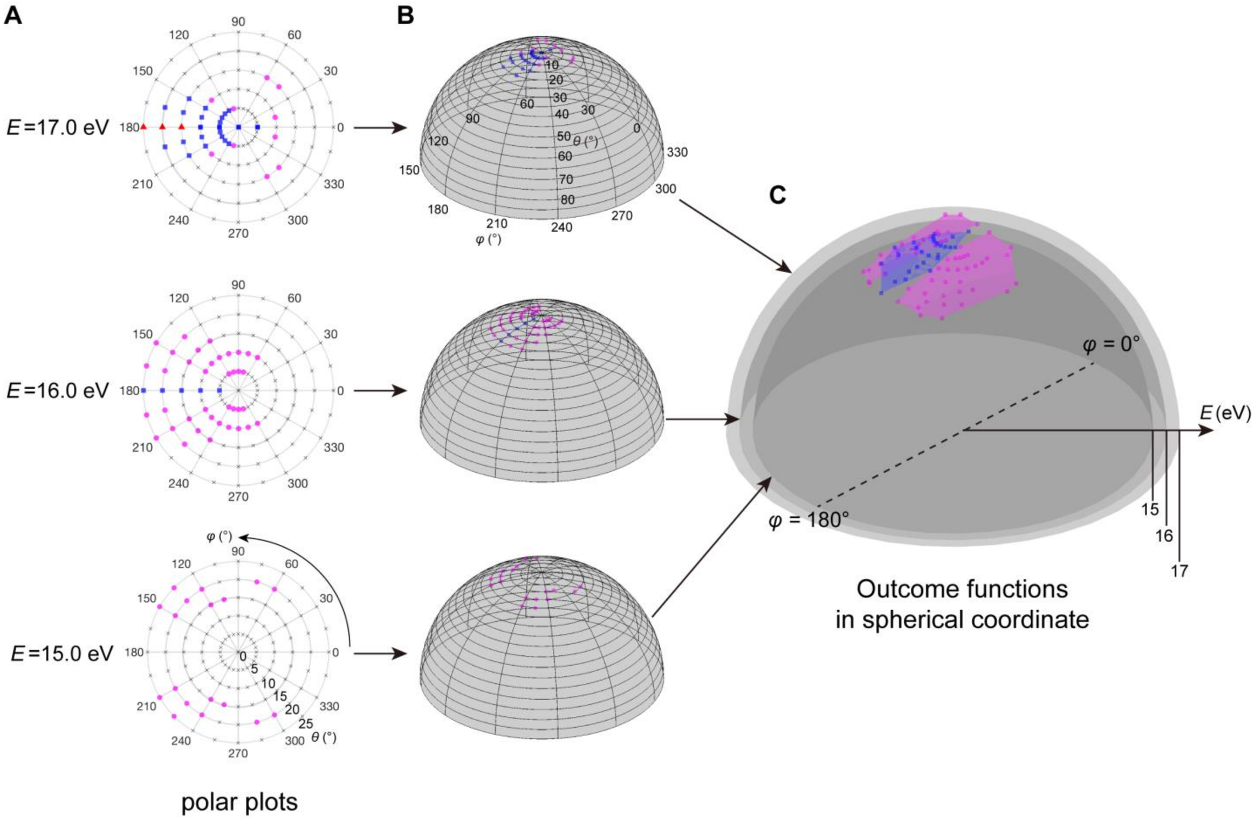

Fig. S5. Construction of PKS. (A) The polar plots of the distribution of dynamic processes with different C atom initial energies (15, 16, and 17 eV). **(B)** The dynamic processes of different energies mapped onto hemispheres, and **(C)** combined into the PKS. Only blue squares (direct exchange) and magenta circles (SW transition) are shown.

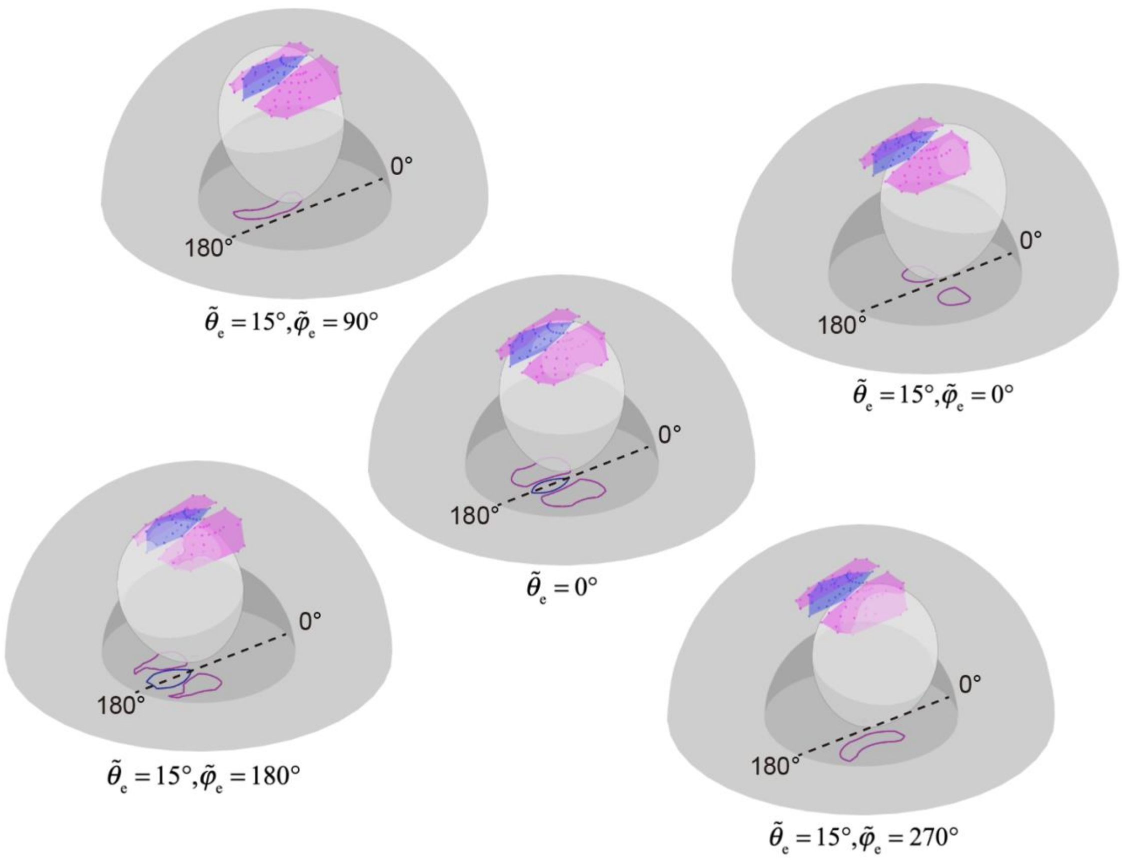

Fig. S6. Selective dynamics by tilting beam. Five different electron incident angles are shown ($\tilde{\theta}_e$ = 0°, and $\tilde{\varphi}_e$ = 0°, 90°, 180°, 270° when $\tilde{\theta}_e$ = 15°). The intersections of the ovoids with different dynamic areas are projected to the bottom surface for a better view. These intersections represent the effective angles for the momentum transferred to the C atom to achieve a certain dynamic process. By tilting the sample or the incident electron beam, different dynamic processes can be selectively initiated.

The generalized *f* with respect to arbitrary electron incident angles ($\tilde{\theta}_e, \tilde{\varphi}_e$) and energy $\tilde{E}_e$ can be written as

$$f(\theta,\varphi,\tilde{\Gamma};\tilde{\Gamma}_e) = E_{max}(\sin\tilde{\theta}_e \cos\tilde{\varphi}_e \cos\varphi \sin\theta + \sin\tilde{\theta}_e \sin\tilde{\varphi}_e \sin\varphi \sin\theta + \cos\tilde{\theta}_e \cos\theta)^2 + \Delta E_{vib}(\theta,\varphi,\tilde{\Gamma}) \tag{S10}$$

where the first term accounts for the ovoid without vibration ($\tilde{E} = 0$), and the second is a correction to the first term by considering the pre-collision status of PKA ($\tilde{\Gamma}$). The above equation (S10) can be simplified for head-on collision ($\tilde{\theta}_e = 0$) and $\tilde{E} = 0$

$$f(\theta,\varphi,\tilde{E}=0;\tilde{\theta}_{\mathrm{e}}=0)=E_{\max}\cos^2\theta \tag{S11}$$

Section S6. Ovoid modification by atom vibration (Doppler amplification effect)

Let us consider the most generic picture of a two-body collision between an electron and an atom (fig. S7A). Again, by writing down the conservation of momentum

$$\left|\tilde{\vec{p}}_{\mathrm{e}}\right|+\left|\tilde{\vec{p}}\right|\cos\alpha=\left|\vec{p}\right|\cos\theta+\left|\vec{p}_{\mathrm{e}}\right|\cos\psi \tag{S12-1}$$

$$\left|\tilde{\vec{p}}\right|\sin\alpha=\left|\vec{p}\right|\sin\theta+\left|\vec{p}_{\mathrm{e}}\right|\sin\psi \tag{S12-2}$$

and the conservation of energy

$$\tilde{E}_{\mathrm{e}}+\tilde{E}=E+E_{\mathrm{e}} \tag{S13}$$

and by inserting equations (S4) and (S5), we get the relation

$$\begin{aligned}&\frac{1}{c}\sqrt{2\tilde{E}_{\mathrm{e}}E_0+\tilde{E}_{\mathrm{e}}^2}+\sqrt{2M\tilde{E}\cos^2\alpha}=\\&\sqrt{2ME}\cos\theta\pm\frac{1}{c}\sqrt{2\tilde{E}_{\mathrm{e}}E_0+\tilde{E}_{\mathrm{e}}^2}\left[1-\frac{c^2}{2\tilde{E}_{\mathrm{e}}E_0+\tilde{E}_{\mathrm{e}}^2}\left(\sqrt{2ME}\sin\theta-\sqrt{2M\tilde{E}\sin^2\alpha}\right)^2\right]^{1/2}\end{aligned} \tag{S14}$$

To simplify the picture, let us only consider an atom vibrating in the same direction as the electron before collision, in which case $a=0$. By solving for E with respect to the angle q, we now get

$$\sqrt{E}=\left[\sqrt{\frac{2\tilde{E}_{\mathrm{e}}E_0+\tilde{E}_{\mathrm{e}}^2}{2Mc^2}}+\sqrt{\tilde{E}}\right]\cos\theta\pm\sqrt{\left[\sqrt{\frac{2\tilde{E}_{\mathrm{e}}E_0+\tilde{E}_{\mathrm{e}}^2}{2Mc^2}}+\sqrt{\tilde{E}}\right]^2\cos^2\theta-\frac{\sqrt{2M\tilde{E}(2\tilde{E}_{\mathrm{e}}E_0+\tilde{E}_{\mathrm{e}}^2)}+M\tilde{E}c}{Mc}} \tag{S15}$$

where the valid range of θ is defined by

$$\left[\sqrt{\frac{2\tilde{E}_{\mathrm{e}}E_0+\tilde{E}_{\mathrm{e}}^2}{2Mc^2}}+\sqrt{\tilde{E}}\right]^2\cos^2\theta-\frac{\sqrt{2M\tilde{E}(2\tilde{E}_{\mathrm{e}}E_0+\tilde{E}_{\mathrm{e}}^2)}+M\tilde{E}c}{Mc}\geq 0 \qquad \text{(S16)}$$

By considering an initial atom vibrational energy $\tilde{E}$ in the range [0, 1] eV, we get Fig. 4B.

The effect of in-plane vibration to the final transferrable energy can be rationalized by an observation frame-translation effect. Consider pre-collisional velocity $\tilde{\boldsymbol{v}}$ and post-collisional velocity $\boldsymbol{v}$ of the PKA. We expect that, order-of-magnitude wise, $M\tilde{\boldsymbol{v}}\cdot\tilde{\boldsymbol{v}}/2\sim 0.1$ eV and $M\boldsymbol{v}\times\boldsymbol{v}/2\sim 10$ eV, where M is the mass of the PKA. Thus $\boldsymbol{v}$ is about 10× the size of $\tilde{\boldsymbol{v}}$. Let us first consider a reference case of $\tilde{\boldsymbol{v}}=0$ and solve for $\boldsymbol{v}$ º $\boldsymbol{v}_0$ for a given impact parameter b, and then "turn on" finite but small $\Delta\tilde{\boldsymbol{v}}$ Since a 60 keV electron moves with the speed 1.3377×10^8 m/s (about 45% of ligh speed), and $\Delta\tilde{\boldsymbol{v}}$ is only of the order 10^3 m/s, seen in a translating frame of velocity $\Delta\tilde{\boldsymbol{v}}$ with respect to the lab frame, the incoming electron velocity is barely changed (60keV→59.999keV), so for the same impact geometry, the outgoing PKA velocity in this co-translating frame is nearly $\boldsymbol{v}_0$. Yet, when transforming back from the translating frame to the lab frame, we need to add back $\boldsymbol{v}_0\rightarrow\boldsymbol{v}_0+\Delta\tilde{\boldsymbol{v}}$, so we obtain

$$\begin{aligned} E_0\equiv\frac{M\boldsymbol{v}_0\cdot\boldsymbol{v}_0}{2} \;&\rightarrow\; E\approx\frac{M(\boldsymbol{v}_0+\Delta\tilde{\boldsymbol{v}})\cdot(\boldsymbol{v}_0+\Delta\tilde{\boldsymbol{v}})}{2} \\ =\frac{M\boldsymbol{v}_0\cdot\boldsymbol{v}_0}{2}+\frac{M\Delta\tilde{\boldsymbol{v}}\cdot\Delta\tilde{\boldsymbol{v}}}{2}&+M\boldsymbol{v}_0\cdot\Delta\tilde{\boldsymbol{v}}=E_0+\tilde{E}+M\boldsymbol{v}_0\cdot\Delta\tilde{\boldsymbol{v}} \end{aligned} \qquad \text{(S17)}$$

Thus from the second term, we get a baseline sensitivity of 1 (if PKA's pre-collision energy is 0.1 eV, this part will be inherited directly), but the third term can give a much larger sensitivity of 10× due to the larger magnitude of $\boldsymbol{v}$ compared to the size of $\tilde{\boldsymbol{v}}$, and this amplification sensivity is also directional (10× or -10× if parallel or anti-parallel fluctuation, 0× if transverse fluctuations) like in Doppler effect.

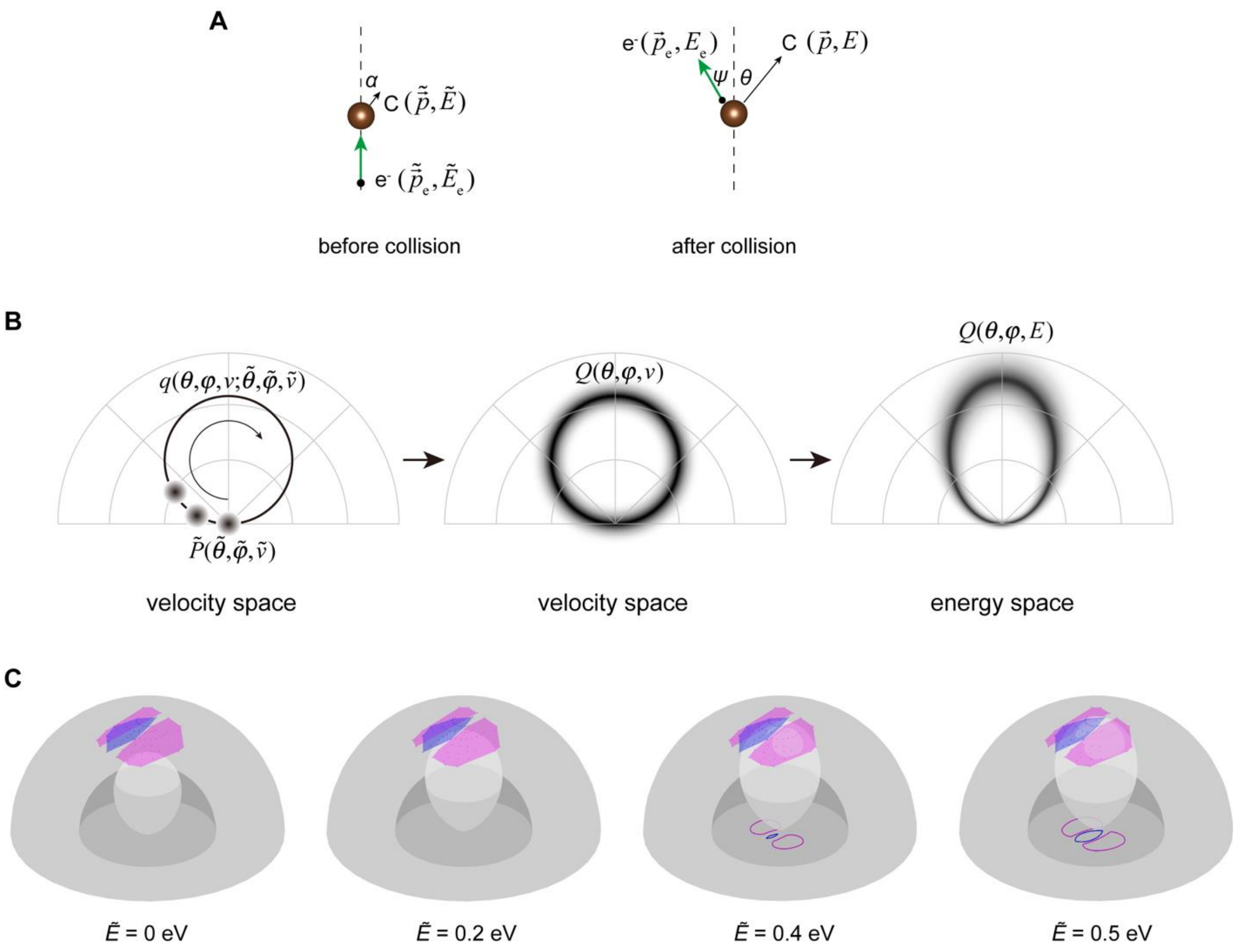

Fig. S7. Ovoid modification by vibration. (A) A schematic illustration of a generic collision between an electron and a C atom. The annotations used in derivation are marked alongside the particles. **(B)** A schematic process of the construction of $Q(G)$. $\tilde{P}$, the angle-resolved vibrational velocity distribution of PKA, is a point spread function that convolutes with each point on q. **(C)** The intersection between the ovoid and PKS evolves with atomic vibration energy, with intersections corresponding to four examples in the range of $\tilde{E} = 0$ to 0.5 eV. Note that a weighed superposition of these ovoid with respect to its probability yields Q shown in (B).

Section S7. Atomic engineering: Manipulation decision tree

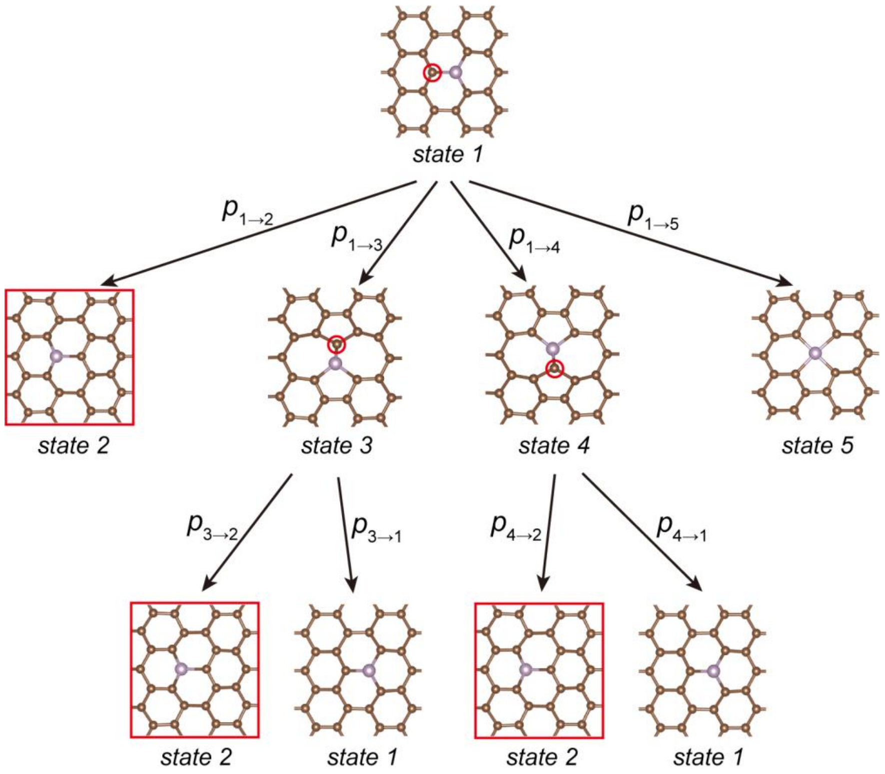

Fig. S8. Decision tree for atomic engineering. $p_{i\to k}$ stands for the probability of a dynamic process from an initial configuration i to final configuration k. We have assumed that the electron incident angles θ_e and φ_e are fixed throughout the whole operation. The state outlined in red indicates the final desired state. Red circles indicate the target atoms of for the electron irradiation.

The probability of each dynamic process can be obtained as

$$p_{i\to k}(\tilde{\Gamma}_e) = \frac{\sigma_{i\to k}(\tilde{\Gamma}_e)}{\sum_k \sigma_{i\to k}(\tilde{\Gamma}_e)} \tag{S18}$$

We can therefore maximize the probability of a specific configuration change by choosing a combination of angles that maximizes the probability of desired branches while minimizing that of undesired ones.

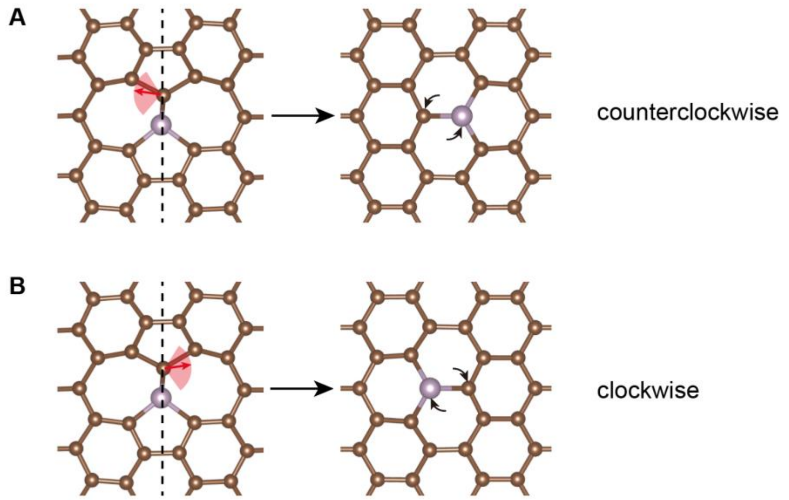

Fig. S9. Selective dynamics from 55-77 structure back to honeycomb. The rotation direction of PKA follows the direction of electron beam, where the **(A)** counterclockwise and **(B)** clockwise rotations are triggered when electron beam is pointing to the left domain and right domain respectively. The shaded red areas is a schematic indication of the available angles for triggering the dynamics, which change with respect to E_C. This selective dynamics applies to both P and Si dopants. Only the clockwise rotation (B) occurs in Fig. 4H of the main text.

Section S8. Method of calculating experimental cross section

The numbers in Table 1 is obtained by the following procedure. The relation between event probability p (with a unit of [# events / s]) and the cross section σ (with a unit of [m]$^{-2}$) is

$$p = s\,j \tag{S18}$$

where j is the electron flux with a unit of [# electrons / m^2 / s]. The probability of events can be obtained from

$$p = 1/t \tag{S19}$$

where t is the duration time of electron irradiation for the event, which is counted starting from the last event. The electron flux j is estimated from the electron current I by the measurement

$$j = I/e/A \tag{S20}$$

where $e = 1.6\times10^{-19}$ C, and A is the effective area of probe size, which can be found in Ref. 2.
